# Bis(η^5^-cyclo­penta­dien­yl)bis­(2,4,6-tri­methyl­phenyl­tellurolato)zirconium(IV)

**DOI:** 10.1107/S1600536808009574

**Published:** 2008-04-16

**Authors:** Andrew L. Hector, William Levason, Gillian Reid, Stuart D. Reid, Michael Webster

**Affiliations:** a School of Chemistry, University of Southampton, Southampton SO17 1BJ, England

## Abstract

The structure of the title compound, [Zr(C_5_H_5_)_2_(C_9_H_11_Te)_2_], consists of a zirconium(IV) centre bonded to two η^5^-coord­inated cyclo­penta­dienyl groups and two mesityltellurolate ligands; the discrete mol­ecule has crystallographic twofold rotation symmetry. The structural parameters compared with those in [(η^5^-Me_5_Cp)_2_Zr(TePh)_2_] [Howard, Trnka & Parkin (1995 ▶). *Inorg. Chem.* **34**, 5900–5909] show that the greater steric demands of the bulky mesityl substituents are accommodated by widening Te—Zr—Te (∼8°) and by more acute Zr—Te—C (∼5°) angles, although the Zr—Te distances are essentially the same. The crystal studied exhibited some inversion twinning.

## Related literature

For a review, see: Arnold (1995 ▶). For related structures, see: Christou *et al.* (1993 ▶); Hector *et al.* (2008 ▶); Howard *et al.* (1995 ▶); Sato & Yoshida (1974 ▶).
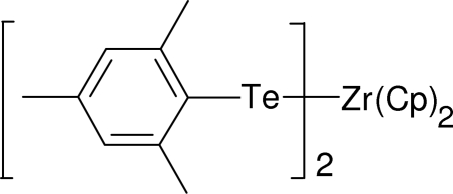


## Experimental

### 

#### Crystal data

[Zr(C_5_H_5_)_2_(C_9_H_11_Te)_2_]
*M* *_r_* = 714.96
Orthorhombic, 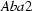

*a* = 9.0483 (15) Å
*b* = 21.881 (6) Å
*c* = 13.806 (4) Å
*V* = 2733.3 (12) Å^3^
*Z* = 4
Mo *K*α radiation
μ = 2.51 mm^−1^
*T* = 120 (2) K
0.20 × 0.10 × 0.02 mm

#### Data collection

Bruker Nonius KappaCCD diffractometer
Absorption correction: multi-scan (*SADABS*; Sheldrick, 2007 ▶) *T*_min_ = 0.758, *T*_max_ = 0.951
9201 measured reflections
3046 independent reflections
2444 reflections with *I* > 2σ(*I*)
*R*_int_ = 0.059

#### Refinement

*R*[*F* ^2^ > 2σ(*F* ^2^)] = 0.040
*wR*(*F* ^2^) = 0.087
*S* = 1.05
3046 reflections
145 parameters
1 restraint
H-atom parameters constrained
Δρ_max_ = 1.50 e Å^−3^
Δρ_min_ = −1.16 e Å^−3^
Absolute structure: Flack (1983 ▶), with 1405 Friedel pairs
Flack parameter: 0.14 (5)

### 

Data collection: *COLLECT* (Hooft, 1998 ▶) and *DENZO* (Otwinowski & Minor, 1997 ▶); cell refinement: *COLLECT* and *DENZO*; data reduction: *COLLECT* and *DENZO*; program(s) used to solve structure: *SHELXS97* (Sheldrick, 2008 ▶); program(s) used to refine structure: *SHELXL97* (Sheldrick, 2008 ▶); molecular graphics: *ORTEPII* (Johnson, 1976 ▶); software used to prepare material for publication: *SHELXL97*.

## Supplementary Material

Crystal structure: contains datablocks I, global. DOI: 10.1107/S1600536808009574/sj2480sup1.cif

Structure factors: contains datablocks I. DOI: 10.1107/S1600536808009574/sj2480Isup2.hkl

Additional supplementary materials:  crystallographic information; 3D view; checkCIF report

## Figures and Tables

**Table:** 

Zr1—C1	2.519 (8)
Zr1—C2	2.519 (7)
Zr1—C3	2.455 (7)
Zr1—C4	2.470 (7)
Zr1—C5	2.504 (8)
Zr1—Te1	2.8694 (10)
Te1—C6	2.150 (7)

**Table:** 

Te1—Zr1—Te1^i^	105.34 (5)
C6—Te1—Zr1	108.06 (16)

**Table:** 

Zr1—Te1—C6—C7	77.0 (5)
Zr1—Te1—C6—C11	−103.8 (5)
Te1^i^—Zr1—Te1—C6	−79.80 (19)

Symmetry code: (i) 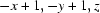.

**Fig. 1.**
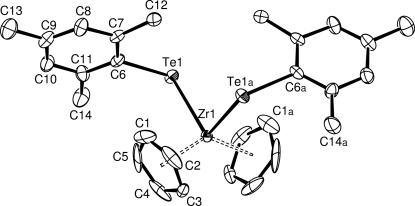
The discrete molecule of (I) showing the atom-labelling scheme. Displacement ellipsoids are drawn at the 50% probability level and H atoms are omitted for clarity. Symmetry operation: a = 1 - x, 1 - y, z.

## supplementary crystallographic information

### Comment

Thiolate ligands (*RS*^-^) form complexes with most metals and metalloids in the Periodic Table. In contrast, much less is known about corresponding selenolates (RSe^-^), and few tellurolate (RTe^-^) complexes have been characterized in detail (Arnold, 1995). The latter include [(η^5^-Me_5_Cp)_2_Zr(TePh)_2_] prepared from [(η^5^-Me_5_Cp)_2_Zr(CO)_2_], PhOH and Ph_2_Te_2_, (Howard *et al.*, 1995) and [(η^5^-Cp)_2_Zr(TePh)_2_] prepared from [(η^5^-Cp)_2_ZrCl_2_] and PhTeLi, (Sato & Yoshida, 1974). We have recently characterized a range of complexes of Ti, Zr and Hf (*M*) of type [(η^5^-Cp)_2_M(Se*^t^*Bu)_2_] and shown that these complexes serve as single-source precursors for LPCVD (low pressure chemical vapour deposition) of MSe_2_ films (Hector *et al.*, 2008), but that the corresponding t-butyltellurolates decompose to deposit elemental tellurium. During attempts to improve the stability of the tellurato-complexes, we obtained crystals of the title complex which we now report.

Red crystals of the title compound (I) were obtained in poor yield by reaction of [(η^5^-Cp)_2_ZrCl_2_] with (Me_3_C_6_H_2_)TeMgBr in anhydrous THF solution. The discrete molecule has 2-fold crystallographic symmetry, and shows the typical metallocene geometry with η^5^-coordinated Cp rings (Zr—C 2.455 (7)–2.519 (8) Å, 2.49 (3) Å (av)) rather shorter than those in [(η^5^-Me_5_Cp)_2_Zr(TePh)_2_] (2.56 (5) Å (av)) (Howard *et al.*, 1995), but similar to those in the silyltellurolate [(η^5^-Cp)_2_Zr{TeSi(SiMe_3_)_3_}_2_] (2.50 (1) Å (av)) (Christou *et al.*, 1993). The Zr—Te distances in [(η^5^-Cp)_2_Zr{TeSi(SiMe_3_)_3_}_2_] (2.866 (1) Å), [(η^5^-Cp)_2_Zr(TeC_6_H_2_Me_3_)_2_] (2.869 (1) Å), and [(η^5^-Me_5_Cp)_2_Zr(TePh)_2_] (2.87 (2) Å) are very similar as are the Te—C distances in the last two compounds (2.150 (7) and 2.12 (2) Å respectively). A more notable difference is in the Te—Zr—Te and Zr—Te—C angles between [(η^5^-Cp)_2_Zr(TeC_6_H_2_Me_3_)_2_] and [(η^5^-Me_5_Cp)_2_Zr(TePh)_2_] with Te—Zr—Te 105.34 (5) ° *versus* 97.2 (1) °, and Zr—Te—C 108.06 (16) ° *versus* 113.1 (7) °, consistent with the greater steric effects of the mesityl groups.

### Experimental

To a stirred suspension of Mg turnings (66 mg, 2.72 mmol) in THF (30 ml) was added MesBr (56 mg, 2.82 mmol). The resulting mixture was stirred for 2 h, after which the Grignard was transferred by cannula to a suspension of freshly ground Te powder (300 mg, 2.35 mmol) in THF (10 ml). The mixture turned orange and was stirred for 1 h. Cp_2_ZrCl_2_ (345 mg, 1.18 mmol) was dissolved in THF (10 ml) and the Grignard solution added dropwise by cannula, during which time the solution turned red. The reaction was allowed to proceed overnight. The volatiles were removed *in vacuo*, the residue extracted with CH_2_Cl_2_ (20 ml) and filtered through celite. The solvent was removed under reduced pressure, the residue crystallized from Et_2_O to produce a small number of red crystals. ^125^Te{^1^H} NMR (CH_2_Cl_2_/CDCl_3_, 300 K): δ_Te_ = 887 p.p.m.

### Refinement

H atoms were placed in calculated positions [C—H = 0.95 (aromatic and Cp) and 0.98 Å (methyl)]. *U*_iso_(H) values for methyl H atoms were set at 1.5*U*_eq_(C) of the bonded C, and the rest at 1.2*U*_eq_(C). Racemic twinning was allowed in the final refinement. The number of Friedel pairs measured is 1405.

### Crystal data

**Table:**

[Zr(C_5_H_5_)_2_(C_9_H_11_Te)_2_]	*F*_000_ = 1376
*M**_r_* = 714.96	*D*_x_ = 1.737 Mg m^−^^3^
Orthorhombic, *A**b**a*2	Mo *K*α radiation λ = 0.71073 Å
Hall symbol: A 2 -2ac	Cell parameters from 7078 reflections
*a* = 9.0483 (15) Å	θ = 2.9–27.5º
*b* = 21.881 (6) Å	µ = 2.51 mm^−^^1^
*c* = 13.806 (4) Å	*T* = 120 (2) K
*V* = 2733.3 (12) Å^3^	Plate, red
*Z* = 4	0.20 × 0.10 × 0.02 mm

### Data collection

**Table:**

Bruker Nonius KappaCCD diffractometer	3046 independent reflections
Radiation source: Bruker-Nonius FR591 rotating anode	2444 reflections with *I* > 2σ(*I*)
Monochromator: graphite	*R*_int_ = 0.059
*T* = 120(2) K	θ_max_ = 27.5º
φ and ω scans	θ_min_ = 3.5º
Absorption correction: multi-scan(SADABS; Sheldrick, 2007)	*h* = −9→11
*T*_min_ = 0.758, *T*_max_ = 0.951	*k* = −28→26
9201 measured reflections	*l* = −17→16

### Refinement

**Table:**

Refinement on *F*^2^	Hydrogen site location: inferred from neighbouring sites
Least-squares matrix: full	H-atom parameters constrained
*R*[*F*^2^ > 2σ(*F*^2^)] = 0.040	*w* = 1/[σ^2^(*F*_o_^2^) + (0.0276*P*)^2^ + 1.8865*P*] where *P* = (*F*_o_^2^ + 2*F*_c_^2^)/3
*wR*(*F*^2^) = 0.087	(Δ/σ)_max_ = 0.001
*S* = 1.05	Δρ_max_ = 1.50 e Å^−^^3^
3046 reflections	Δρ_min_ = −1.16 e Å^−^^3^
145 parameters	Extinction correction: none
1 restraint	Absolute structure: Flack (1983), 1405 Friedel pairs
Primary atom site location: structure-invariant direct methods	Flack parameter: 0.14 (5)
Secondary atom site location: difference Fourier map	

### Special details

**Table:**

Geometry. All e.s.d.'s (except the e.s.d. in the dihedral angle between two l.s. planes) are estimated using the full covariance matrix. The cell e.s.d.'s are taken into account individually in the estimation of e.s.d.'s in distances, angles and torsion angles; correlations between e.s.d.'s in cell parameters are only used when they are defined by crystal symmetry. An approximate (isotropic) treatment of cell e.s.d.'s is used for estimating e.s.d.'s involving l.s. planes.
Refinement. Refinement of *F*^2^ against ALL reflections. The weighted *R*-factor *wR* and goodness of fit *S* are based on *F*^2^, conventional *R*-factors *R* are based on *F*, with *F* set to zero for negative *F*^2^. The threshold expression of *F*^2^ > σ(*F*^2^) is used only for calculating *R*-factors(gt) *etc*. and is not relevant to the choice of reflections for refinement. *R*-factors based on *F*^2^ are statistically about twice as large as those based on *F*, and *R*- factors based on ALL data will be even larger.

### Fractional atomic coordinates and isotropic or equivalent isotropic displacement parameters (Å^2^)

**Table:**

	*x*	*y*	*z*	*U*_iso_*/*U*_eq_	
Zr1	0.5000	0.5000	−0.06403 (8)	0.01613 (16)	
Te1	0.74620 (4)	0.522546 (15)	0.06201 (7)	0.02158 (12)	
C1	0.4387 (14)	0.6120 (4)	−0.0508 (8)	0.066 (3)	
H1	0.4389	0.6355	0.0070	0.079*	
C2	0.3184 (8)	0.5854 (4)	−0.0922 (6)	0.043 (2)	
H2	0.2208	0.5869	−0.0670	0.052*	
C3	0.3574 (7)	0.5607 (3)	−0.1807 (6)	0.0324 (16)	
H3	0.2915	0.5453	−0.2284	0.039*	
C4	0.5054 (9)	0.5768 (3)	−0.1952 (7)	0.051 (2)	
H4	0.5578	0.5726	−0.2544	0.062*	
C5	0.5545 (11)	0.6072 (4)	−0.1164 (10)	0.067 (4)	
H5	0.6475	0.6271	−0.1110	0.081*	
C6	0.7316 (6)	0.6154 (3)	0.1121 (5)	0.0222 (14)	
C7	0.6309 (7)	0.6307 (3)	0.1845 (4)	0.0221 (16)	
C8	0.6241 (7)	0.6926 (3)	0.2140 (6)	0.0278 (15)	
H8	0.5545	0.7039	0.2624	0.033*	
C9	0.7141 (8)	0.7370 (4)	0.1756 (6)	0.0293 (19)	
C10	0.8146 (8)	0.7201 (3)	0.1041 (6)	0.0312 (17)	
H10	0.8773	0.7502	0.0764	0.037*	
C11	0.8248 (6)	0.6597 (3)	0.0723 (6)	0.0261 (14)	
C12	0.5339 (7)	0.5842 (3)	0.2340 (5)	0.0296 (17)	
H12A	0.4787	0.5611	0.1852	0.044*	
H12B	0.4644	0.6052	0.2771	0.044*	
H12C	0.5955	0.5562	0.2719	0.044*	
C13	0.6987 (9)	0.8020 (4)	0.2084 (7)	0.041 (2)	
H13A	0.5976	0.8161	0.1961	0.062*	
H13B	0.7685	0.8278	0.1727	0.062*	
H13C	0.7198	0.8046	0.2779	0.062*	
C14	0.9425 (8)	0.6443 (4)	−0.0014 (6)	0.0388 (19)	
H14A	0.8958	0.6352	−0.0638	0.058*	
H14B	0.9984	0.6085	0.0207	0.058*	
H14C	1.0097	0.6791	−0.0088	0.058*	

### Atomic displacement parameters (Å^2^)

**Table:**

	*U*^11^	*U*^22^	*U*^33^	*U*^12^	*U*^13^	*U*^23^
Zr1	0.0179 (3)	0.0137 (3)	0.0168 (3)	0.0028 (3)	0.000	0.000
Te1	0.02085 (18)	0.01997 (19)	0.0239 (2)	0.00485 (15)	−0.00464 (18)	−0.0039 (2)
C1	0.139 (10)	0.024 (4)	0.035 (7)	0.037 (5)	−0.038 (7)	−0.002 (4)
C2	0.033 (4)	0.041 (5)	0.055 (7)	0.022 (3)	0.023 (4)	0.028 (4)
C3	0.049 (4)	0.014 (3)	0.035 (4)	−0.002 (3)	−0.028 (4)	0.003 (3)
C4	0.065 (5)	0.039 (4)	0.050 (6)	0.032 (4)	0.032 (6)	0.034 (4)
C5	0.060 (6)	0.016 (5)	0.126 (11)	−0.014 (4)	−0.053 (7)	0.028 (6)
C6	0.022 (3)	0.022 (3)	0.022 (4)	0.006 (2)	−0.004 (3)	−0.006 (3)
C7	0.023 (3)	0.026 (4)	0.017 (4)	0.002 (3)	−0.003 (2)	−0.008 (3)
C8	0.024 (3)	0.023 (4)	0.036 (4)	0.003 (3)	−0.002 (3)	−0.009 (3)
C9	0.025 (4)	0.031 (5)	0.032 (5)	−0.002 (3)	−0.002 (3)	−0.013 (4)
C10	0.032 (4)	0.028 (4)	0.033 (5)	−0.007 (3)	0.003 (3)	−0.007 (3)
C11	0.019 (3)	0.027 (3)	0.032 (4)	−0.003 (2)	0.002 (3)	0.002 (3)
C12	0.037 (4)	0.028 (4)	0.023 (4)	−0.002 (3)	0.002 (3)	−0.004 (3)
C13	0.039 (4)	0.030 (5)	0.055 (6)	0.003 (4)	0.001 (4)	−0.013 (4)
C14	0.033 (4)	0.044 (5)	0.040 (5)	−0.010 (3)	0.016 (3)	−0.015 (4)

### Geometric parameters (Å, °)

**Table:**

Zr1—C1	2.519 (8)	C5—H5	0.9500
Zr1—C2	2.519 (7)	C6—C7	1.393 (9)
Zr1—C3	2.455 (7)	C6—C11	1.398 (9)
Zr1—C4	2.470 (7)	C7—C8	1.415 (9)
Zr1—C5	2.504 (8)	C7—C12	1.507 (9)
Zr1—C3^i^	2.455 (7)	C8—C9	1.375 (11)
Zr1—C4^i^	2.470 (7)	C8—H8	0.9500
Zr1—C5^i^	2.504 (8)	C9—C10	1.393 (11)
Zr1—C2^i^	2.519 (7)	C9—C13	1.499 (12)
Zr1—C1^i^	2.519 (8)	C10—C11	1.395 (9)
Zr1—Te1	2.8694 (10)	C10—H10	0.9500
Zr1—Te1^i^	2.8694 (10)	C11—C14	1.512 (9)
Te1—C6	2.150 (7)	C12—H12A	0.9800
C1—C2	1.360 (13)	C12—H12B	0.9800
C1—C5	1.390 (15)	C12—H12C	0.9800
C1—H1	0.9500	C13—H13A	0.9800
C2—C3	1.382 (12)	C13—H13B	0.9800
C2—H2	0.9500	C13—H13C	0.9800
C3—C4	1.399 (10)	C14—H14A	0.9800
C3—H3	0.9500	C14—H14B	0.9800
C4—C5	1.350 (13)	C14—H14C	0.9800
C4—H4	0.9500		
			
C3—Zr1—C3^i^	98.0 (4)	C2—C1—C5	107.3 (9)
C3—Zr1—C4^i^	82.9 (3)	C2—C1—Zr1	74.3 (5)
C3^i^—Zr1—C4^i^	33.0 (2)	C5—C1—Zr1	73.3 (6)
C3—Zr1—C4	33.0 (2)	C2—C1—H1	125.9
C3^i^—Zr1—C4	82.9 (3)	C5—C1—H1	125.9
C4^i^—Zr1—C4	85.7 (5)	Zr1—C1—H1	125.9
C3—Zr1—C5^i^	102.3 (4)	C1—C2—C3	109.6 (7)
C3^i^—Zr1—C5^i^	53.6 (2)	C1—C2—Zr1	74.3 (4)
C4^i^—Zr1—C5^i^	31.5 (3)	C3—C2—Zr1	71.3 (4)
C4—Zr1—C5^i^	115.4 (5)	C1—C2—H2	125.1
C3—Zr1—C5	53.6 (2)	C3—C2—H2	125.1
C3^i^—Zr1—C5	102.3 (4)	Zr1—C2—H2	125.1
C4^i^—Zr1—C5	115.4 (5)	C2—C3—C4	105.8 (7)
C4—Zr1—C5	31.5 (3)	C2—C3—Zr1	76.4 (4)
C5^i^—Zr1—C5	146.4 (6)	C4—C3—Zr1	74.1 (4)
C3—Zr1—C2^i^	130.0 (3)	C2—C3—H3	126.3
C3^i^—Zr1—C2^i^	32.2 (3)	C4—C3—H3	126.3
C4^i^—Zr1—C2^i^	52.8 (2)	Zr1—C3—H3	126.3
C4—Zr1—C2^i^	112.2 (3)	C5—C4—C3	108.9 (8)
C5^i^—Zr1—C2^i^	52.3 (3)	C5—C4—Zr1	75.6 (5)
C5—Zr1—C2^i^	121.5 (3)	C3—C4—Zr1	72.9 (4)
C3—Zr1—C2	32.2 (3)	C5—C4—H4	125.2
C3^i^—Zr1—C2	130.0 (3)	C3—C4—H4	125.2
C4^i^—Zr1—C2	112.2 (3)	Zr1—C4—H4	125.2
C4—Zr1—C2	52.8 (2)	C4—C5—C1	108.3 (8)
C5^i^—Zr1—C2	121.5 (3)	C4—C5—Zr1	72.9 (5)
C5—Zr1—C2	52.3 (3)	C1—C5—Zr1	74.5 (6)
C2^i^—Zr1—C2	162.2 (4)	C4—C5—H5	125.6
C3—Zr1—C1^i^	133.6 (3)	C1—C5—H5	125.6
C3^i^—Zr1—C1^i^	53.5 (3)	Zr1—C5—H5	125.6
C4^i^—Zr1—C1^i^	52.8 (3)	C7—C6—C11	120.6 (6)
C4—Zr1—C1^i^	135.3 (3)	C7—C6—Te1	119.9 (5)
C5^i^—Zr1—C1^i^	32.1 (3)	C11—C6—Te1	119.5 (5)
C5—Zr1—C1^i^	152.7 (3)	C6—C7—C8	117.7 (6)
C2^i^—Zr1—C1^i^	31.3 (3)	C6—C7—C12	123.0 (6)
C2—Zr1—C1^i^	151.2 (3)	C8—C7—C12	119.3 (6)
C3—Zr1—C1	53.5 (3)	C9—C8—C7	122.6 (7)
C3^i^—Zr1—C1	133.6 (3)	C9—C8—H8	118.7
C4^i^—Zr1—C1	135.3 (3)	C7—C8—H8	118.7
C4—Zr1—C1	52.8 (3)	C8—C9—C10	118.2 (7)
C5^i^—Zr1—C1	152.7 (3)	C8—C9—C13	119.9 (7)
C5—Zr1—C1	32.1 (3)	C10—C9—C13	121.8 (8)
C2^i^—Zr1—C1	151.2 (3)	C9—C10—C11	121.2 (7)
C2—Zr1—C1	31.3 (3)	C9—C10—H10	119.4
C1^i^—Zr1—C1	171.7 (5)	C11—C10—H10	119.4
C3—Zr1—Te1	135.49 (14)	C10—C11—C6	119.6 (6)
C3^i^—Zr1—Te1	94.75 (19)	C10—C11—C14	118.1 (6)
C4^i^—Zr1—Te1	125.22 (16)	C6—C11—C14	122.3 (6)
C4—Zr1—Te1	108.2 (2)	C7—C12—H12A	109.5
C5^i^—Zr1—Te1	119.3 (3)	C7—C12—H12B	109.5
C5—Zr1—Te1	82.0 (2)	H12A—C12—H12B	109.5
C2^i^—Zr1—Te1	73.43 (16)	C7—C12—H12C	109.5
C2—Zr1—Te1	118.2 (2)	H12A—C12—H12C	109.5
C1^i^—Zr1—Te1	87.3 (3)	H12B—C12—H12C	109.5
C1—Zr1—Te1	87.7 (3)	C9—C13—H13A	109.5
C3—Zr1—Te1^i^	94.75 (19)	C9—C13—H13B	109.5
C3^i^—Zr1—Te1^i^	135.49 (14)	H13A—C13—H13B	109.5
C4^i^—Zr1—Te1^i^	108.2 (2)	C9—C13—H13C	109.5
C4—Zr1—Te1^i^	125.22 (16)	H13A—C13—H13C	109.5
C5^i^—Zr1—Te1^i^	82.0 (2)	H13B—C13—H13C	109.5
C5—Zr1—Te1^i^	119.3 (3)	C11—C14—H14A	109.5
C2^i^—Zr1—Te1^i^	118.2 (2)	C11—C14—H14B	109.5
C2—Zr1—Te1^i^	73.43 (16)	H14A—C14—H14B	109.5
C1^i^—Zr1—Te1^i^	87.7 (3)	C11—C14—H14C	109.5
C1—Zr1—Te1^i^	87.3 (3)	H14A—C14—H14C	109.5
Te1—Zr1—Te1^i^	105.34 (5)	H14B—C14—H14C	109.5
C6—Te1—Zr1	108.06 (16)		
			
Zr1—Te1—C6—C7	77.0 (5)	Te1^i^—Zr1—Te1—C6	−79.80 (19)
Zr1—Te1—C6—C11	−103.8 (5)		

Symmetry codes: (i) −*x*+1, −*y*+1, *z*.
